# Social networks and cognitive function in older adults: findings from the HAPIEE study

**DOI:** 10.1186/s12877-021-02531-0

**Published:** 2021-10-18

**Authors:** Yifan Nie, Marcus Richards, Ruzena Kubinova, Anastasiya Titarenko, Sofia Malyutina, Magdalena Kozela, Andrzej Pajak, Martin Bobak, Milagros Ruiz

**Affiliations:** 1 Research Department of Epidemiology and Public Health, University College London, 1-19 Torrington Place, London, WC1E 7HB UK; 2 MRC Unit for Lifelong Health and Ageing at UCL, University College London, London, UK; 3 Centre for Environmental Health Monitoring, National Institute of Public Health, Prague, Czech Republic; 4 Research Institute of Internal and Preventive Medicine, Branch of the Federal Research Centre Institute of Cytology and Genetics, Siberian Branch of Russian Academy of Medical Sciences, Novosibirsk, Russia; 5 Institute of Public Health, Faculty of Health Sciences, Jagiellonian University Medical College, Krakow, Poland; 6 Research Centre for Toxic Compounds in the Environment, Faculty of Sciences, Masaryk University, Brno, Czech Republic; 7 Faculty of Physical Education and Sport, Charles University, Prague, Czech Republic

**Keywords:** Ageing, Czech Republic, Cognitive function, Cognitive decline, Poland, Russia, Social networks, Social relationships

## Abstract

**Background:**

Social networks are associated with better cognitive health in older people, but the role of specific aspects of the social network remains unclear. This is especially the case in Central and Eastern Europe. This study examined associations between three aspects of the social network (network size of friends and relatives, contact frequency with friends and relatives, and social activity participation) with cognitive functions (verbal memory, learning ability, verbal fluency, processing speed, and global cognitive function) in older Czech, Polish, and Russian adults.

**Methods:**

Linear regression estimated associations between baseline social networks and cognitive domains measured at both baseline and follow-up (mean duration of follow-up, 3.5 ± 0.7 years) in 6691 participants (mean age, 62.2 ± 6.0 years; 53.7% women) from the Health, Alcohol and Psychosocial factors In Eastern Europe (HAPIEE) study.

**Results:**

Cross-sectional analyses, adjusted for country, age, and sex, showed positive associations of global cognitive function with social activity participation and network size of friends and relatives, but not with contact frequency in either network. Further adjustment for sociodemographic, behavioural, and health characteristics attenuated the associations with network size of relatives (P-trend = 0.074) but not with network size of friends (P-trend = 0.036) or social activities (P-trend< 0.001). In prospective analyses, network size and social activity participation were also linked with better cognition in simple models, but the associations were much stronger for social activities (P-trend< 0.001) than for network size of friends (P-trend = 0.095) and relatives (P-trend = 0.425). Adjustment for baseline cognition largely explained the prospective associations with network size of friends (P-trend = 0.787) and relatives (P-trend = 0.815), but it only slightly attenuated the association with social activities (P-trend< 0.001). The prospective effect of social activities was largely explained by sociodemographic, health behavioural, and health covariates (P-trend = 0.233). Analyses of specific cognitive domains generally replicated the cross-sectional and prospective findings for global cognitive function.

**Conclusions:**

Older Central and Eastern European adults with larger social networks and greater social activities participation had better cognitive function, but these associations were stronger at baseline than over the short-term follow-up.

**Supplementary Information:**

The online version contains supplementary material available at 10.1186/s12877-021-02531-0.

## Background

In recent decades, population ageing has become a worldwide pressing issue. A top concern for healthy ageing is the maintenance of cognitive health which is essential for older adults’ well-being and quality of life [1]. The Lancet Commission on Dementia Prevention, Intervention and Care has concluded that social isolation is among the top five modifiable risk factors in adults aged ≥65 years which can reduce dementia incidence [1]. As adults grow older, social relationships endure various changes including the migration of children, family members, and friends; a dwindling number of social network members due to death or infirmity; and individual factors such as declining states of health which can inhibit social engagement [2]. Social isolation refers to the objective physical estrangement from social networks, described as the system of social relationships surrounding an individual (“social network structure”) and the characteristics of those relations (“quality of network ties”) [3]. Social network structure refers to the size, range, and homogeneity of network members, as well as the boundedness between members through kinship or neighbourhood. Quality of network ties is reflected by the frequency of organisational participation, as well as the level of contact, reciprocity, and social support between members, among other attributes [3].

Emerging evidence shows that social networks independently predict cognitive functioning over and above socio-economic circumstances, health behaviours, and physical health. According to a meta-analysis of 19 cohort studies, individuals with weak social networks, characterized by small network size or low social activity participation, had 1.08 higher odds of cognitive impairment [4]. Additional analyses assessing network size and social activity separately found that both were independently protective against cognitive decline. Moreover, eight studies using a composite measure of social networks and social support reported that adults with low scores had 1.12 higher odds of cognitive decline. However, the prospective effect of social networks on cognition may be overestimated given apparent publication bias or reverse causation in studies with relatively short-term follow-up [4]. A systematic review of 39 studies found that overall, social activity was associated with better global cognitive function, executive function, working memory, and processing speed, but not with episodic memory or attention [5]. Social networks, measured by network size and contact frequency, were associated with global cognitive function, but not with specific cognitive domains. However, the results were inconsistent between studies due to differences in target population, study design, sample size, and measurement of social networks [5] .

Hypothesised mechanisms are that active social networks help build neural pathways and cognitive reserve, bestow resilience against the neuropathology of dementia, alleviate damaging stress, and foster a healthier lifestyle [6]. Among these, the cognitive reserve hypothesis has gained much attention with respect to interpersonal interaction as a modifiable risk factor for dementia [7]. Preserving an active social network can protect against cognitive decline by imparting cognitive stimulation and thereby promoting cognitive reserve. Unlike other organs where cells activation results in “wear and tear”, cognitive stimulation provided through social networks can protect the brain from cognitive impairment by activating nerve cells in a physiological range [8]. Cognitive reserve reflects how well people with brain damage can use undamaged parts of the brain to compensate for existing pathology. Apart from social relationships, evidence indicates that cognitive reserve is affected by a range of educational and occupational activities throughout the life course [9].

Despite the strong growing evidence on the relationship between social networks and cognitive functioning, several gaps must be addressed. Since most studies were conducted in the US and Northwestern Europe, evidence from Central and Eastern Europe (CEE) is very limited [4, 5]. Older CEE adults are an invaluable population in which to examine the relationship between social networks and cognitive function for several reasons. Firstly, social networks are embedded in the larger socio-cultural context and conditioned by upstream factors, such as the political transition in CEE [3]. Individuals living through rapid socio-cultural changes often endure an absence of stable social relationships and interpersonal resources [10]. The post-communist transition had profound ramifications for the fabric of social networks among older CEE adults [11]. Compared to other Europeans, older CEE adults have reported smaller network sizes [12]; lower quality networks, measured by the level of expected social support from network members; and less frequent organisational participation [13]. Secondly, a critical issue is that social networks may hold a distinct role in countries characterised by a family-based welfare model with low levels of public responsibility. As older CEE adults receive less social care from the state compared to those from other European countries with more generous social transfers, social networks may be vital in this context [14]. Thirdly, social relationships, heavily influenced by prevailing cultural norms, are likely different between individualistic and collectivist societies [15]. Expressed familial obligation for older relatives is generally stronger in CEE than in Western Europe [11]. These considerations are important for social networks as a resource for health in CEE, which has the highest level of dementia-related disability in Europe [16]. Notably, Berkman and colleagues remarked that very few studies on social networks and health [3], including cognition [4, 5], have considered the consistency of this relationship across different macro-social contexts whereby networks are formed and sustained in culturally specific ways [3].

Moreover, previous studies including systematic reviews and meta-analyses have examined associations either between specific aspects of the social network with global cognitive function or pathological cognitive disorders [2, 17, 18], or between summary measures of the social network with specific cognitive domains [4, 5]. A few recent studies have comprehensively examined associations between specific social network characteristics and different cognitive domains [19, 20], so far in Swedish and US Chinese older adults.

Using data from a population-based prospective study of ageing in the Czech Republic, Poland, and Russia, this study investigated the cross-sectional and prospective associations between social network size, frequency of contact with social network members and social activity participation with global cognitive function and four specific cognitive domains.

## Methods

### Study population

The Health, Alcohol and Psychosocial factors In Eastern Europe (HAPIEE) project is a multi-centre prospective cohort study in Russia (Novosibirsk), Poland (Krakow), the Czech Republic (six medium-sized towns), and Lithuania (Kaunas) [21]. The present study used data from the Russian, Polish, and Czech arms of the study. Register-based random samples of 28,945 adults aged 45 to 69 years old were recruited at baseline (2002–2005). Individual response rates were 61% in Poland (*n* = 10,728) and Russia (*n* = 9360); and 55% (*n* = 8857) in the Czech Republic. The first follow-up assessment took place in 2006–2008 whereby 59% (*n* = 5097), 62% (*n* = 6721), and 66% (*n* = 6417) of the original Czech, Polish, and Russian cohort samples were successfully re-examined [22]. Years of follow-up varied between Czech (mean = 3.6; SD = 0.5), Polish (mean = 4.0; SD = 0.4), and Russian (mean = 3.1; SD = 0.7) participants.

### Cognitive function

At baseline, cognitive assessments were performed on all non-working participants and an approximately 20% random subsample of working participants. At follow-up, cognitive function was assessed in all participants. Four cognitive tests were measured in the following order: immediate word recall, animal naming task, letter cancellation task and delayed word recall. An audio recording of a 10-word list was played to participants over three consecutive 1-min trials. Immediate word recall was measured using the total number of correctly recalled words from each 1-min trial (range 0–30). The animal naming task involved asking participants to name as many different animals as possible within 1 min. Every correctly named animal was summed to derive a total score. The letter cancellation task instructed participants to cross out the letters “P” and “W” (P and Ш in Russia) from a grid of randomly chosen letters as accurately as possible within 1 min (range 0–65). Delayed word recall was assessed by the number of words correctly recalled after an approximately five-minute interval, during which other cognitive tests were administered (range 0–10). Respectively, the immediate word recall, delayed word recall, animal naming [23], and letter cancellation [24] tasks measure verbal memory, learning ability, verbal fluency [23], and processing speed [24]. These cognitive measures have been widely validated to detect expected age-related changes in cognition and more pronounced impairment associated with neurogenerative conditions [23, 25].

To facilitate comparability, the four cognitive test scores were standardized as z-scores (mean = 0; SD = 1) using the population means and standard deviations observed at each time point. Although cognitive performance between individuals varies by age and sex, for example, cognitive data were normalised using a regression-based approach which adjusted for these demographic characteristics. This method has proven reliable when analysing cognition data over a maximum of two measurement occasions [26]. A measure of global cognitive function was generated using the arithmetic average of the four individual z-scores. Composite measures of global cognitive function have been similarly derived from brief neuropsychological tests in previous studies [27], and are strong predictors of Alzheimer’s disease [28] and dementia-related neuropathologic lesions [29]. The psychometric quality of global cognitive function in the HAPIEE study has been previously demonstrated [22, 30].

### Social networks

Social networks were assessed at baseline. Network size of friends and relatives was based on the following two questions: “How many friends do you see at least once a week?” and “How many relatives who do not live in your household do you see at least once a week?” Responses were collected on a 4-point scale ranging from “none”, “1 or 2”, “3 to 5”, and “more than 5” times per week.

Contact frequency with friends and relatives was measured using the following two questions: “How often do you visit friends?” and “Are you regularly in contact with your relatives who do not live in your household?” Responses were collected on a 6-point scale ranging from “several times a week”, “about once a week”, “several times a month”, “about once a month”, “less than once a month” to “I do not have friends outside relatives” or “I do not have relatives/no relatives outside of my household.”

Participants were also asked “Are you a member of a club or organisation (e.g., sports club, church, political party)?” using a “yes” / “no” option and “How often do you participate in these activities?” using a 5-point scale ranging from “several times a week”, “several times a month”, 3 “about once a month” 4 “several times a year” 5 “never or almost never”. Individuals who responded no to the first question were classified as “never or almost never” participating in social activities.

The six questions on social networks were adapted from the Berkman-Syme Social Network Index, an 11-item self-reported questionnaire designed to assess the social connectedness of individuals and to identify those likely to be socially isolated [10]. Although baseline data on contact frequency and social activity participation were collected in all three countries, relevant data on network size were collected in Poland and Russia, but not in the Czech Republic.

### Covariates

Baseline covariates included country, age, sex, education (primary or lower, vocational, secondary, university), number of household amenities (0–12) (based on a list of 12 common items including microwave, colour television, washing machine), partnered/not partnered, smoking status (current, occasional, former, never) alcohol drinking frequency (ranging from “never” to “five times or more per week”), alcohol intake (grams), physical activity based on the number of hours during a typical week, self-rated health (very poor or poor, fair, good or very good), number of chronic diseases (0–4) based on previous heart attack or acute myocardial infarction, angina or ischemic heart disease, stroke, and cancer), and depressive symptoms measured using the Center for Epidemiologic Studies Depression 20-item Scale (0–60). Using data on economic activity, work status classified participants as working (employed, entrepreneur/owner of a company, farmer, re-employed pensioner) or not working (housewife, unemployed pensioner, unemployed).

### Statistical analysis

**Fig. 1:**
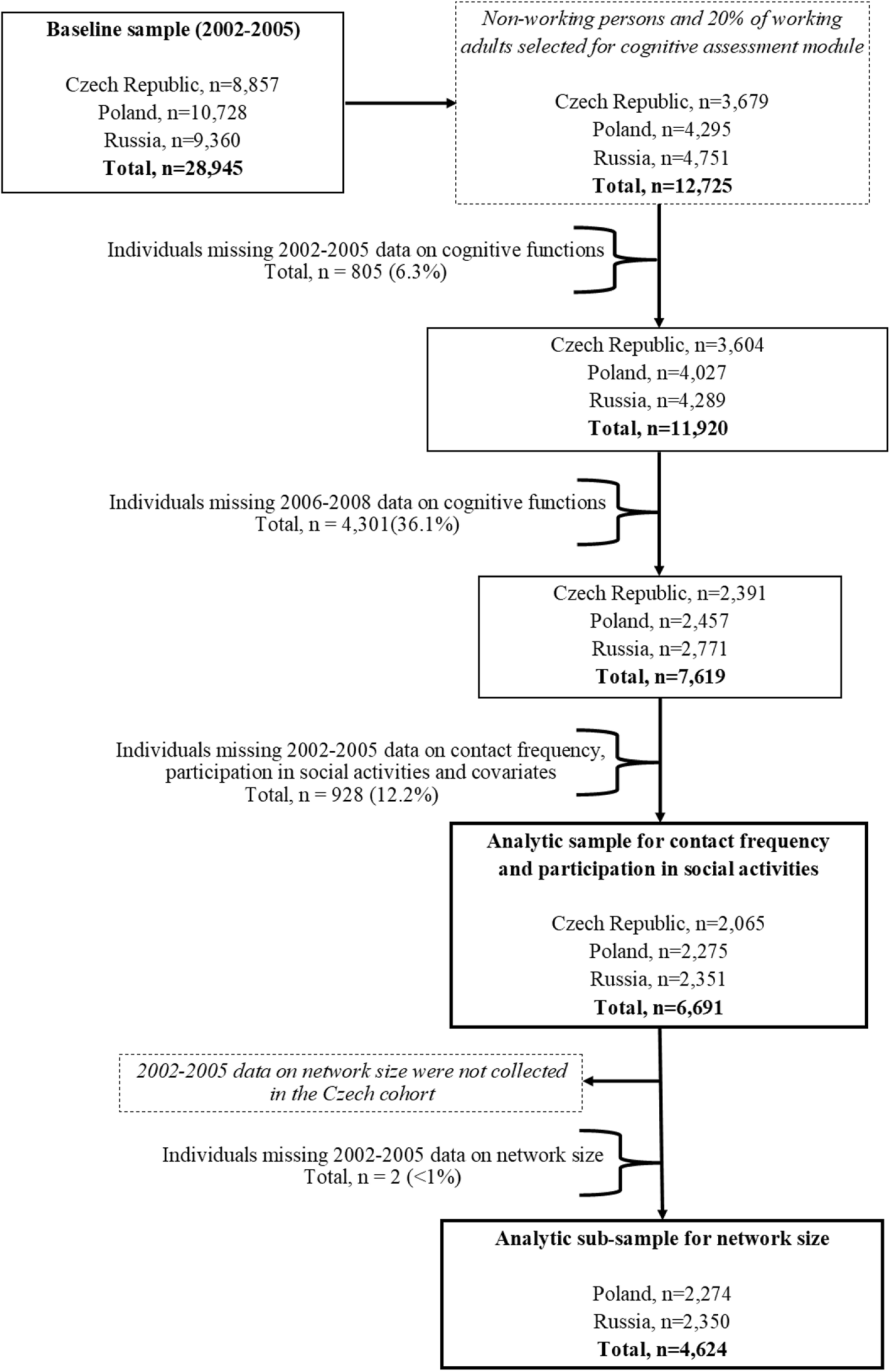
Flow chart of inclusion and exclusion criteria for the HAPIEE analytic samples

All non-working participants and a 20% random sample of working individuals (*n* = 12,725) were selected for cognitive assessment at baseline; 93.7% (*n* = 11,920) had complete data on all four cognitive domains. Of these participants, 63.9% (*n* = 7619) took part in the cognitive re-assessment during the follow-up investigation. Czech, Polish, and Russian participants with complete data across all cognitive domains at both time points plus complete data on contact frequency and social activities and covariates comprised the main analytic sample (*n* = 6691). Since data on network size were not collected in the Czech Republic, an analytic sub-sample consisting of 4624 Polish and Russian participants was derived for these specific analyses. Full inclusion and exclusion criteria used to form the analytic samples are detailed in a flowchart (Fig. 1).

Linear regression models were used to measure the cross-sectional and prospective associations between markers of social networks and z-scores of cognitive domains. Cross-sectional and prospective associations were tested using a nested approach that first adjusted for country, age, and sex; then additionally for the remaining covariates. Prospective associations were also adjusted for each respective cognitive z-score at baseline in an additional intermediate model. Stepwise models were repeated to test for linear trend in the hypothesised associations by treating ordinal exposures as continuous variables. Effect modification was tested by including two-way interaction terms between exposures on one hand and country, age, sex, education, and work status on the other hand, but there was no evidence of heterogeneity by these covariates below 0.10 significance level.

Sensitivity checks were performed to assess whether the complete case analyses suffered from selection and attrition biases between baseline and follow-up. Firstly, the representativeness of the complete prospective samples (*n* = 6691 and *n* = 4624) was evaluated by comparing study characteristics with all participants with cognition data at baseline (*n* = 11,920). Secondly, to examine whether the complete case findings underestimated associations between social network characteristics and global cognitive function at baseline; the country, age, and sex-adjusted cross-sectional associations were re-calculated in participants with baseline data on cognition, contact frequency and social activities (*n* = 10,440) and network size (*n* = 7365) and covariates.

Given the variability in the duration of follow-up from baseline to re-examination in the analytic samples (Additional file 1), we repeated the cross-sectional and prospective analyses of global cognitive function restricted to participants who were re-examined between 3 and 4 years of follow-up to assess whether this variability may have influenced the main findings.

Since the analyses examined five social networks characteristics, correction for multiple testing was applied to the cross-sectional and prospective associations between exposures and global cognitive function. Bonferroni-corrected *p*-values were calculated to assess the risk of Type 1 or false positive error which can result from multiple comparisons.

All analyses were conducted in Stata V.15.

## Results

Table 1 reports the participant characteristics in the pooled sample and separately by country. At baseline and follow-up, Czech adults generally performed better on cognitive tests than Polish and Russian individuals. Among participants with data on network size, social networks of friends and relatives were larger for Polish than Russian adults. On the other hand, older adults in Poland were less likely to see their friends and relatives about once a week or several times a week compared to those in Czechia and Russia. Social activities participation was most frequent in Czechia, followed by Poland then Russia. Country differences in cognitive function and social network characteristics are further detailed in Additional file 2.

**Table 1:** Study characteristics of the HAPIEE analytic samples

	Total^a^ *n* = 6691	Czech Republic (*n* = 2065)	Poland (*n* = 2275)	Russia (*n* = 2351)
% or Mean (SD) for follow-up measures (2006/2008)				
Immediate recall (0–30)	22.0 (4.2)	23.0 (3.7)	20.4 (4.3)	22.7 (4.0)
Delayed recall (0–10)	7.6 (2.0)	7.7 (1.8)	6.8 (2.0)	8.1 (1.8)
Verbal fluency	22.5 (7.1)	23.6 (6.4)	20.9 (6.0)	22.9 (8.3)
Processing speed (0–65)	17.3 (5.1)	17.8 (5.0)	16.6 (5.2)	17.6 (5.0)
% or Mean (SD) for baseline measures (2002/2005)				
Immediate recall (0–30)	20.8 (4.3)	22.8 (3.4)	19.0 (3.8)	20.5 (4.5)
Delayed recall (0–10)	7.0 (1.9)	7.7 (1.7)	6.7 (1.8)	6.7 (2.0)
Verbal fluency	20.6 (6.9)	23.6 (6.6)	19.4 (5.8)	19.1 (7.2)
Processing speed (0–65)	17.7 (5.5)	18.2 (4.5)	17.6 (6.4)	17.4 (5.3)
Age (years)	62.2 (6.0)	61.6 (6.1)	64.3 (4.1)	60.5 (6.7)
Female	53.7%	54.6%	48.9%	57.6%
Network size of friends^b^				
None	46.5%		29.6%	62.8%
1 or 2	35.3%	Not available	51.1%	19.9%
3 to 5	14.3%		14.8%	13.7%
More than 5	4.0%		4.5%	3.5%
Network size of relatives^b^				
None	40.5%		31.6%	49.1%
1 or 2	35.3%		43.1%	27.8%
3 to 5	20.6%		21.2%	20.0%
More than 5	3.6%		4.1%	3.1%
Contact frequency with friends				
No friends	6.7%	1.8%	7.0%	10.6%
Less than once a month	26.7%	20.2%	31.5%	28.0%
About once a month	21.7%	22.8%	21.8%	20.5%
Several times a month	16.3%	24.0%	17.1%	8.6%
About once a week	16.4%	20.2%	14.9%	14.6%
Several times a week	12.2%	11.0%	7.7%	17.7%
Contact frequency with relatives				
No relatives	2.6%	0.8%	3.4%	3.3%
Less than once a month	18.6%	8.9%	26.4%	19.5%
About once a month	14.1%	9.5%	18.9%	13.6%
Several times a month	14.5%	16.6%	18.8%	8.5%
About once a week	24.5%	27.3%	18.4%	27.9%
Several times a week	25.8%	36.9%	14.2%	27.3%
Participation in social activities				
Never or not a member	81.8%	68.0%	86.7%	89.2%
At least several times a year	8.8%	15.2%	6.8%	5.0%
Several times a month or more	9.4%	16.8%	6.5%	5.8%
Educational level				
Primary or lower	9.6%	10.9%	12.6%	5.4%
Vocational	26.2%	35.7%	18.2%	25.4%
Secondary	37.0%	38.5%	39.0%	33.7%
University	27.3%	14.9%	30.2%	35.5%
Number of household amenities (0–12)	6.3 (2.2)	6.8 (2.2)	6.1 (2.1)	6.0 (2.2)
Not working	64.6%	62.6%	78.8%	52.3%
Not partnered	25.6%	23.5%	25.1%	27.9%
Smoking status				
Current	20.4%	19.5%	21.0%	20.7%
Former	25.2%	30.6%	31.5%	14.5%
Never	54.3%	49.9%	47.5%	64.8%
Alcohol drinking frequency				
Never	21.5%	11.7%	38.3%	13.7%
< 1/month	29.9%	24.3%	23.4%	41.0%
1–3/month	20.3%	22.2%	18.0%	20.8%
1–4/week	21.9%	27.5%	16.8%	22.0%
More than 5/week	6.4%	14.2%	3.4%	2.5%
Alcohol intake per week (grams)	2580.7 (6134.5)	3902.2 (7940.0)	1323.6 (3764.2)	2636.6 (5914.7)
Physical activity per week (hours)	21.2 (14.3)	19.5 (15.4)	19.5 (13.2)	24.2 (13.8)
Self-rated health				
Very poor or poor	14.5%	8.2%	14.1%	20.3%
Fair	59.6%	49.4%	57.0%	71.2%
Very good or good	26.0%	42.5%	28.9%	8.6%
Number of chronic diseases (0–4)	3.7 (0.6)	3.8 (0.5)	3.5 (0.7)	3.7 (0.6)
CES-D 20 score (0–60)	10.9 (7.9)	9.3 (7.9)	10.6 (7.6)	12.4 (7.9)

^a^Table estimates are based on the analytic sample for frequency of contact with social network members and participation in social activities (*n* = 6691), with exception to baseline frequencies on social network size which was estimated using the sub-sample with these data (*n* = 4624)

^b^As data on social network size were not collected in the Czech Republic, a sub-sample was used for the analyses on social network size using data from Poland and Russia (*n* = 4624)

### Cross-sectional associations between social networks and cognitive function

Country, age, and sex-adjusted associations at baseline indicated a dose response pattern between larger social networks of friends (P-trend = 0.003) and relatives (P-trend = 0.010) as well as more frequent social activities (P-trend< 0.001) with higher global cognitive function (Table 2). These stepwise associations were reduced by sociodemographic characteristics, health behaviours, and health, but remained strong for network size of friends (P-trend = 0.036) and social activities (P-trend< 0.001) after full adjustment.

**Table 2:** Linear regression coefficients (95% CIs) for cross-sectional associations of social network characteristics with global cognitive function

Social network measure	Model 1^a^		Model 2^b^	
	b	95% CI	b	95% CI
**Network size of friends**				
None	0.01	− 0.05, 0.06	0.02	-0.03, 0.07
1 or 2	Reference		Reference	
3 to 5	0.08	0.01, 0.15	0.08	0.01, 0.14
More than 5	0.18	0.06, 0.30	0.13	0.02, 0.24
*P-trend*	0.003		0.036	
**Network size of relatives**				
None	0.00	-0.05, 0.05	0.01	−0.04, 0.06
1 or 2	Reference		Reference	
3 to 5	0.04	−0.02, 0.11	0.05	0.00, 0.11
More than 5	0.16	0.03, 0.28	0.12	0.01, 0.24
*P-trend*	0.010		0.074	
**Contact frequency with friends**				
No friends	−0.12	− 0.20, − 0.05	0.01	− 0.06, 0.09
Less than once a month	Reference		Reference	
About once a month	0.06	0.01, 0.12	0.04	−0.01, 0.08
Several times a month	0.02	−0.03, 0.08	0.01	−0.05, 0.06
About once a week	0.01	−0.04, 0.07	0.03	−0.03, 0.08
Several times a week	−0.03	− 0.10, 0.03	0.01	−0.05, 0.07
*P-trend*	0.490		0.655	
**Contact frequency with relatives**				
No relatives	−0.01	− 0.13, 0.11	− 0.01	− 0.13, 0.10
Less than once a month	Reference		Reference	
About once a month	−0.02	− 0.08, 0.05	0.00	−0.06, 0.06
Several times a month	0.10	0.04, 0.17	0.09	0.03, 0.15
About once a week	0.05	−0.01, 0.11	0.06	0.00, 0.11
Several times a week	0.05	−0.01, 0.11	0.03	−0.03, 0.08
*P-trend*	0.017		0.091	
**Participation in social activities**				
Never or not a member	Reference		Reference	
At least several times a year	0.19	0.12, 0.25	0.07	0.01, 0.13
Several times a month or more	0.22	0.16, 0.29	0.10	0.04, 0.16
*P-trend*	< 0.001		< 0.001	

^a^Adjusted for country, age and sex

^b^Adjusted for country, age, sex, education, household amenities, work status, marital status, smoking status, alcohol drinking frequency, alcohol intake, physical activity, self-rated health, number of chronic diseases and depressive symptoms

Cross-sectional associations between contact frequency with friends and relatives and global cognitive function at baseline were weaker and not as linear. Counter to theory, greater contact frequency with friends was not associated with better global cognitive function, before (P-trend = 0.490) or after (P-trend = 0.655) adjustment. There was some evidence of dose response for contact frequency with relatives (P-trend = 0.017); however, only the moderate group who contacted relatives several times a month had higher global cognitive function (0.10, 95% CI 0.04, 0.17) (Model 1). Furthermore, the sparse effects for contact with relatives were attenuated after full adjustment (P-trend = 0.091).

Cross-sectional associations between social network characteristics and specific cognitive domains further highlighted the importance of having a larger friend network and engaging in more social activities (Additional file 3). For example, network size of friends was associated with stepwise increases in immediate recall, verbal fluency, and processing speed; while more frequent social activities were linked with higher levels of cognition across all cognitive domains including delayed recall.

### Prospective associations between social networks and cognitive function

Compared to the baseline results, country, age, and sex-adjusted associations between network size of friends (P-trend = 0.095) and relatives (P-trend = 0.425) with global cognitive function were less substantial over the follow-up (Table 3). For instance, the Model 1 results suggested that adults with more than 5 friends and relatives had higher global cognitive function by 0.06 (95% CI − 0.05, 0.17) and 0.08 (95% CI − 0.03, 0.19) units, in turn, but prospectively these effects were much weaker. The benefits associated with having more than 5 friends (− 0.02, 95% CI -0.11, 0.08) and relatives (0.01, 95% CI -0.09, 0.11) were fully attenuated by baseline cognition (Model 2).

**Table 3:** Linear regression coefficients (95% CIs) for prospective associations of social network characteristics with global cognitive function

Social network measure	Model 1^a^		Model 2^b^		Model 3^c^	
	b	95% CI	b	95% CI	b	95% CI
**Network size of friends**						
None	− 0.02	− 0.07, 0.03	− 0.02	− 0.07, 0.02	− 0.03	−0.07, 0.02
1 or 2	Reference		Reference		Reference	
3 to 5	0.01	−0.05, 0.08	−0.03	− 0.08, 0.03	−0.01	− 0.07, 0.04
More than 5	0.06	−0.05, 0.17	−0.02	− 0.11, 0.08	−0.03	− 0.12, 0.06
*P-trend*	0.095		0.787		0.643	
**Network size of relatives**						
None	0.00	−0.05, 0.05	0.00	−0.04, 0.04	0.00	−0.04, 0.04
1 or 2	Reference		Reference		Reference	
3 to 5	0.01	−0.05, 0.07	−0.01	− 0.06, 0.04	0.00	− 0.05, 0.04
More than 5	0.08	−0.03, 0.19	0.01	−0.09, 0.11	0.00	−0.09, 0.10
*P-trend*	0.425		0.815		0.828	
**Contact frequency with friends**						
No friends	−0.13	−0.20, − 0.06	−0.07	− 0.13, − 0.01	−0.01	− 0.07, 0.05
Less than once a month	Reference		Reference		Reference	
About once a month	0.06	0.01, 0.11	0.02	−0.02, 0.06	0.02	−0.02, 0.06
Several times a month	0.00	−0.05, 0.05	−0.02	− 0.07, 0.02	−0.02	− 0.06, 0.03
About once a week	0.02	−0.03, 0.07	0.00	−0.04, 0.05	0.03	−0.02, 0.07
Several times a week	−0.04	−0.10, 0.02	− 0.03	−0.08, 0.02	0.01	−0.04, 0.06
*P-trend*	0.580		0.827		0.535	
**Contact frequency with relatives**						
No relatives	−0.01	−0.12, 0.10	− 0.01	−0.11, 0.08	− 0.01	−0.10, 0.08
Less than once a month	Reference		Reference		Reference	
About once a month	−0.07	−0.13, − 0.01	−0.06	− 0.11, − 0.01	−0.05	− 0.10, − 0.01
Several times a month	0.03	− 0.03, 0.09	−0.02	− 0.07, 0.13	−0.01	− 0.06, 0.03
About once a week	−0.04	− 0.09, 0.01	−0.06	− 0.11, − 0.02	−0.05	− 0.09, − 0.01
Several times a week	0.00	− 0.04, 0.06	−0.02	− 0.06, 0.03	−0.03	− 0.07, 0.01
*P-trend*	0.583		0.405		0.203	
**Participation in social activities**						
Never or not a member	Reference		Reference		Reference	
At least several times a year	0.15	0.09, 0.21	0.06	0.01, 0.11	0.01	−0.04, 0.06
Several times a month or more	0.19	0.13, 0.25	0.08	0.03, 0.13	0.03	−0.02, 0.08
*P-trend*	< 0.001		< 0.001		0.233	

^a^Adjusted for country, age and sex

^b^Adjusted for country, age, sex and baseline global cognitive function

^c^Adjusted for country, age, sex, baseline global cognitive function, education, household amenities, work status, marital status, smoking status, alcohol drinking frequency, alcohol intake, physical activity, self-rated health, number of chronic diseases and depressive symptoms

Otherwise, the prospective findings mirrored the cross-sectional findings in several ways. The prospective results confirmed the weak role of contact frequency with friends (P-trend = 0.580) and relatives (P-trend = 0.583) on the one hand, and the strong beneficial effects of social activities (P-trend< 0.001) on the other hand (Model 1). While higher levels of global cognitive function for adults participating in social activities “at least several times a year” and “several times a month or more” were partially attenuated by baseline cognition to 0.06 (95% CI 0.01, 0.11) and 0.08 (95% CI 0.03, 0.13) units, respectively, the overall graded association remained strong (P-trend< 0.001) (Model 2). The ensuing cognitive benefits related with social activities were explained by differences in socioeconomic circumstances, health behaviours, and physical and mental health (P-trend = 0.233) (Model 3).

Prospective associations between social networks and specific cognitive domains also emphasized differences between the impact of social activities versus network size and contact frequency (Additional file 4). Adults with more frequent social activities had higher levels of immediate recall (P-trend = 0.002), delayed recall (P-trend< 0.001), verbal fluency (P-trend = 0.012), and processing speed (P-trend< 0.001) after adjusting for country, age, sex and baseline function. In contrast, there was no stepwise pattern between network size nor contact frequency with any cognitive domain, except for limited evidence linking a larger network of friends with better delayed recall (P-trend = 0.038).

### Sensitivity analyses

Despite some participant attrition from baseline to follow-up, there were very minor differences in baseline cognitive functions between complete and incomplete cases (Additional file 5). Complete cases, however, had slightly stronger social networks, were more socioeconomically advantaged and reported better self-rated health as well as fewer depressive symptoms. Despite these observed differences between samples, the country, age, and sex-adjusted cross-sectional associations in participants with available data on global cognitive function, social networks, and covariates at baseline (Additional file 6) were overwhelmingly like those observed in the complete prospective sample (Table 2). At the very minimum, the cross-sectional findings do not appear to be underestimated by the exclusion of participants lost to follow-up.

Up to 16% of participants from the analytic samples had their second cognitive assessment before or after the 3–4-year period. Cross-sectional and prospective analyses restricted to participants who were followed up between 3 to 4 years yielded results essentially identical to those of the main analyses (Additional files 7 and 8).

Cognitive benefits associated with larger network sizes, which were only apparent cross-sectionally, did not survive after adjustment for multiple testing (Additional files 9 and 10). For example, Bonferroni corrected *p*-values of the fully adjusted associations indicating higher cognitive function among adults who saw 3 to 5 friends per week or more than 5 friends per week, respectively, were 0.401 and 0.412. The results for participation in social activities, however, appeared more robust. Cross-sectional and prospective associations adjusted for country, age, and sex were statistically significant below 0.001 after Bonferroni correction. While adjustment for Model 2 covariates diminished the Bonferroni adjusted statistical significance associated with moderate social activity participation (i.e., several times a year) in cross-sectional (*p*-value = 0.075) and prospective (*p* = 0.320) analyses, high activity participation (i.e., several times a month or more) remained associated with higher cognitive function at baseline (*p*-value = 0.016) and follow-up (*p*-value = 0.004).

## Discussion

### Summary of findings

This large-scale population-based investigation in the Czech Republic, Poland, and Russia found that older adults with larger network sizes and greater social activity participation had better cognitive function at baseline. Social activities, in particular, continued to have a favourable effect on cognition after 3–4 years of follow-up. Sociodemographic factors, health behaviours, and physical and mental health partially attenuated cross-sectional associations, but fully accounted for the prospective associations. Overall, analyses of specific cognitive domains confirmed the cross-sectional and prospective findings for global cognitive function.

### Comparison with previous literature

Overall, the positive cross-sectional associations between network size and social activity participation with global cognitive function align with previous studies [17, 19, 20]. Yet, our prospective findings revealed that social activity participation, but not network size, was beneficial for cognitive function. While this contradicts a recent systematic review which found that both measures predicted better global cognitive function, memory, and executive function, irrespective of the length of follow-up [31], another up-to-date appraisal of long-term (> 10 years) studies found that social activity participation had a greater protective influence on dementia risk than network size [32]. The prospective influence of social activities was explained by individual baseline characteristics in our study. This is consistent with two studies of longer duration which found that incident dementia was not reduced by social activities (nor by contact frequency with friends and family) in older English adults [6, 33], one of which observed a constriction of social networks in the prodromal stage of dementia [6]. Although social networks decline as dementia progresses [6], it was found that elderly people with Alzheimer’s disease who had larger network sizes maintained higher working memory and semantic memory [34].

Network size and activity participation predicted better cognitive function, but effect sizes were stronger at baseline than at follow-up. Thus, it is essential to consider reverse causality since maintaining strong social networks require some degree of cognitive capacity [35]. Previous studies provide mixed evidence on the direction of association. A Swedish study found that network size was linked with better subsequent episodic memory, semantic memory, and visuospatial ability, but baseline performance of these cognitive functions did not predict future network size over a 5-year period [20]. Yet, an Australian study identified a bidirectional association between social network size and similar cognitive domains over a 6-year period, suggesting a complex interplay between social relationships and cognitive function in later life [36]. These bidirectional hypotheses, however, do not clarify whether stronger social networks protect against cognitive decline by bestowing greater cognitive reserve or whether the ability to sustain social relationships is a marker of cognitive reserve and capacity. Taken together, these studies emphasise that only limited conclusions can be drawn from our cross-sectional and short-term prospective findings.

Despite evidence on the cognitive benefits of social networks broadly measured, the present study found strongest empirical support for activity participation, which was consistent in cross-sectional and prospective analyses on global cognitive function, verbal memory, learning ability, verbal fluency, and processing speed. Participation in formal group activities may provide richer integration with the wider community beyond more proximal connections with friends and relatives [37]. Therefore, the cognitive gains associated with these activities may be greater than network size and contact frequency because they more effectively reduce social isolation. Taking part in communal activities may also increase opportunities to maintain existing contacts and establish new social ties, which is often difficult in later life [4, 5, 18]. Social activities may also instill greater cognitive reserve and enhanced cognitive function because they provide stimulating opportunities to interact with broader, more diverse communal ties and execute certain skills and tasks with others [8, 9]. The cognitive benefits associated with these activities may conceivably vary between social clubs, religious, political, or community organisations. Although the data precluded us from analysing specific activities, the degree to which they are protective will possibly vary by their capacity to enhance cognitive reserve, influence lifestyle factors, provide social support, and help cope with stress [38].

While the prospective benefits of social activities on subsequent cognition were explained by health behaviours, physical and mental health, our study does not address whether these are confounding or mediating factors. While each set of variables may operate as a pathway between social networks and cognitive functioning, path analyses ideally require data from at least three time points. We therefore adjusted for these covariates in our analyses.

### The central and eastern European context

Older adults from post-communist European countries have described smaller, less activity-based, and less socially supportive networks than elsewhere in the region. Although our study confirmed more active social networks in Czechia than Poland and Russia [12, 13], associations were similar between countries. Existing research has largely been conducted in high-income, Western countries [4, 5, 18, 31, 32], but the relationship between social networks and cognition is likely context specific. Several reasons may explain why the hypothesized associations are weaker in CEE. Although social networks are posited to enhance cognitive function through healthier lifestyles, additional analyses (available upon request) found that HAPIEE participants with stronger social networks were more likely to smoke and drink alcohol. Since these behaviours are important risk factors for dementia [1], this may partly explain why social networks did not benefit cognitive function in CEE. As cognitive function appears positively associated with light to moderate alcohol use, but negatively associated with heavy use [7], the role of alcohol may be particularly complex in this context. In collectivist societies, social relationships may be activated to protect adults as they age, especially in CEE where public options for elder support and care are limited [11, 14, 15]. The bidirectional link between social networks and cognition [20, 36] may be weaker in societies with stronger norms of reciprocity and caregiving. As cognition deteriorates, network members may be less prone to disengage when relationships become more difficult to maintain, or be more inclined to provide instrumental social support, including tangible aid and assistance [39].

### Strengths and weaknesses

Several study limitations should be considered. Firstly, although the re-examination of the HAPIEE cohorts was planned to occur between 3 and 4 years of follow-up from baseline, the timing of pilot procedures of the re-examination interview resulted in a shorter 1–2-year interval of follow-up for some participants, while for others, late response bias led to a longer 5–6-year interval of follow-up. While these issues affected a small share of Czech (4.9%) and Polish (4.7%) participants, 26.1% of Russian participants were re-examined outside of the intended period. Sensitivity analyses restricted to participants followed up between 3 to 4 years indicated that the main findings were not affected by the variability in time to follow-up.

Secondly, compared to other prospective studies on social networks and cognition which varied from 10 to 28 years of follow-up [17, 20, 40, 41], our study covered a relatively short-term follow-up period. This weakness could not be avoided as the third cognitive assessment has not taken place in all HAPIEE cohorts. This is an important limitation because the hypothesised effects may be small and accumulate gradually over the longer life course [7]. While several studies have suggested that social networks can reduce the rate of age-related cognitive decline [17, 20, 40, 41], we were unable to perform an analysis of change using data from two measurement occasions. To quantify associations between social networks and subsequent cognitive function, it was appropriate to adjust for baseline function in the prospective analyses to parse apart short-term effects from the contemporaneous effects observed in the cross-sectional analyses. Despite these said shortcomings, the prospective analyses addressed an important weakness of the cross-sectional analyses. Although larger network sizes and greater social activity participation were cross-sectionally linked with higher cognitive function, these associations cannot differentiate cause and effect. Since reverse causation is plausible according to the observational literature [20, 35, 36], this bias is partially addressed by the prospective analyses. Indeed, the prospective findings suggest that reverse causation may explain some of the positive cross-sectional findings, particularly for social network size.

Thirdly, older adults with larger social networks and greater social activities participation had better cognitive function at baseline, but these associations were weaker over the follow-up. This raises the possibility of reverse causality which has been discussed in several studies [6, 36]. Due to limited data availability, we could not exclude participants diagnosed with Alzheimer’s disease or dementia near the time of cognitive assessment. The short study period also precluded us from discarding cognition data from the initial years of follow-up.

Fourthly, additional analyses were conducted on verbal memory, learning ability, verbal fluency, and processing speed, respectively, to determine whether associations varied across cognitive domains. Although the literature has recognised that social networks may help to sustain levels of prospective memory, crystallised ability, and fluid intelligence in middle age and beyond [4, 5, 31], these associations could not be investigated due to data availability. A further implication is that our measure of global cognitive function was limited to four specific domains, while more comprehensive measures were derived in previous studies given data availability on additional cognitive domains [27–29]. While this is an important study limitation, the psychometric quality of global cognitive function in HAPIEE has been previously demonstrated [22, 30]. Self-reported data on social networks may be inaccurate due to social desirability bias which may lead individuals to report higher levels of social connectedness. Older adults with worse cognition may have also been more likely to misreport their existing social networks. As only several studies examined associations between specific social network characteristics and global cognitive function and different cognitive domains [19, 20], the present study aimed to provide comprehensive evidence of these relations in CEE. While this analytic approach appreciates the multidimensional aspects of both social networks and global cognitive function [35], it also raises the issue of multiple comparisons. The present study was motivated by a priori theory and aimed to compare the cognitive gains associated with different aspects of a person’s social network [42], but this approach may increase the risk of Type 1 or false positive error in the findings. Sensitivity analyses for multiple testing suggest that findings for social activity participation were more robust than for social network size, which did not withstand Bonferroni adjustment.

Fifthly, data on hearing impairment were not collected during the main examinations at baseline (2002–2005) and follow-up (2006–2008), so we did not include this covariate in our analyses. Hearing impairment is not only an established risk factor for dementia [1, 7]; but can also impede psychosocial functioning, such as the ability to maintain social ties [41]. In Poland and Russia, data were collected on factors which may have affected performance on cognitive assessment tests. Very few (< 5%) adults reported that hearing problems interfered with cognitive testing. Adjusting for this variable, which likely captured severe hearing loss, did not affect the findings. As this variable was not collected in Czechia, this sensitivity check was not included in the main analysis.

Lastly, participants with weaker social networks, greater socioeconomic disadvantages, worse self-rated health and more depressive symptoms were more likely to withdraw from the study. While sensitivity analyses showed that cross-sectional associations did not appear to be underestimated in the complete prospective sample, it is unknown how prospective associations would have differed with higher response rates over the follow-up. Importantly, baseline cognitive function scores were slightly higher in complete cases than in participants lost to follow-up, which may underestimate the prospective findings.

Aside from these study limitations, data from the HAPIEE study allowed us to perform a population-based study on social networks and cognitive function in community-dwelling older adults from the Czech Republic, Poland, and Russia. Despite the wealth of evidence on social networks and mid-late life cognition, there is a dearth of findings from CEE [4, 5]. While findings of the protective effect of stronger social networks appear consistent in different settings investigated so far, the significance and perception of social isolation vary between societies [7]. As social relationships are embedded and shaped by the larger socio-cultural context, the relationship (and potential mechanisms) between social networks and cognitive health may well be unique for older adults who experienced the unprecedented disruption of social ties due to the political transformation in the region [3].

## Conclusions

In conclusion, older adults from the Czech Republic, Poland, and Russia with larger social networks and greater social activities participation had better cognitive function, but these associations were stronger at baseline than over 3–4 years of follow-up. Future ageing research should examine the potential benefits of social networks on cognition over the longer life course, as well as the socio-biological mechanisms that may underpin the hypothesized associations.

## Supplementary Information

**Additional file 1 **Distribution of years of follow-up in the main analytic sample for contact frequency and participation in social activities (*n*=6,691).

**Additional file 2.** Comparison of cognitive functions and social network characteristics between HAPIEE cohorts.

**Additional file 3.** Additional File 3. Cross-sectional associations of social network characteristics with specific cognitive functions.

**Additional file 4.** Prospective associations of social network characteristics with specific cognitive functions.

**Additional file 5.** Comparison of baseline study characteristics between complete (n=6,691 and n=4,624) and incomplete (n=11,920) cases.

**Additional file 6.** Comparison of country, age, and sex adjusted cross-sectional associations between social network characteristics and global cognition among complete (n=6,691 and n=4,624) and incomplete cases due to loss to follow-up (n=10,440 and 7,365).

**Additional file 7.** Cross-sectional associations of social network characteristics with global cognitive function in participants re-examined between 3-4 years of follow-up.

**Additional file 8.** Prospective associations of social network characteristics with global cognitive function in participants re-examined between 3-4 years of follow-up.

**Additional file 9.** Cross-sectional associations between social network characteristics and global cognitive function with Bonferroni corrected p-values.

**Additional file 10.** Prospective associations between social network characteristics and global cognitive function with Bonferroni corrected p-values.

## Data Availability

The datasets analysed during the current study are not publicly available due to data confidentiality restrictions, but are available from the following co-authors on reasonable request: Prof. Ruzena Kubinova, Prof. Sofia Malyutina, Prof. Andrzej Pajak, and Prof. Martin Bobak.

## Abbreviations

CEE: Central and Eastern Europe
CI: Confidence interval
HAPIEE: Health, Alcohol and Psychosocial factors In Eastern Europe
SD: Standard deviation
