# Supplementary material for: Social networks and cognitive function in older adults: findings from the HAPIEE study
Source: BMC Geriatr. 2021 Oct 18;21:570. doi: 10.1186/s12877-021-02531-0 (PMC8524850; doi:10.1186/s12877-021-02531-0)
Supplement: Supplementary file 1 — Additional file 1 Distribution of years of follow-up in the main analytic sample for contact frequency and participation in social activities (n=6,691). [file 12877_2021_2531_MOESM1_ESM.pdf]

Additional File 1. Distribution of years of follow-up in the main analytic sample for contact frequency and participation in social activities (n=6,691)

Years of follow-up	% (n)			
	Czech Republic	Poland	Russia	Total
1	0	0	0.21 (5)	0.07 (5)
2	4.21 (87)	0	21.52 (506)	8.86 (593)
3	42.76 (883)	7.12 (162)	57.55 (1,353)	35.84 (2,398)
4	52.30 (1,080)	88.13 (2,005)	16.38 (385)	51.86 (3,470)
5	0.73 (15)	4.75 (108)	4.13 (97)	3.29 (220)
6	0	0	0.21 (5)	0.07 (5)
Total % (n)	100 (2,065)	100 (2,275)	100 (2,351)	100 (6,691)
