# Supplementary material for: Social networks and cognitive function in older adults: findings from the HAPIEE study
Source: BMC Geriatr. 2021 Oct 18;21:570. doi: 10.1186/s12877-021-02531-0 (PMC8524850; doi:10.1186/s12877-021-02531-0)
Supplement: Supplementary file 2 — Additional file 2. Comparison of cognitive functions and social network characteristics between HAPIEE cohorts. [file 12877_2021_2531_MOESM2_ESM.pdf]

Additional File 2. Comparison of cognitive functions and social network characteristics between HAPIEE cohorts

	Mean or % of total sample	Mean or % difference from total sample			P-value of country differences*
		Czech Republic	Poland	Russia	
Cognitive function at follow-up (2006/2008)					
Immediate recall (0-30)	22.0	1.0	-1.6	0.7	P<0.001
Delayed recall (0-10)	7.6	0.1	-0.8	0.5	P<0.001
Verbal fluency	22.5	1.1	-1.6	0.4	P<0.001
Processing speed (0-65)	17.3	0.5	-0.7	0.3	P<0.001
Cognitive function at baseline (2002/2005)					
Immediate recall (0-30)	20.8	2.0	-1.8	-0.3	P<0.001
Delayed recall (0-10)	7.0	0.7	-0.3	-0.3	P<0.001
Verbal fluency	20.6	3.0	-1.2	-1.5	P<0.001
Processing speed (0-65)	17.7	0.5	-0.1	-0.3	P<0.001
Social network characteristics at baseline (2002/2005)					
Network size of friends**					P<0.001
None	46.5%		-16.9	16.3	
1 or 2	35.3%		15.8	-15.4	
3 to 5	14.3%		0.5	-0.6	
More than 5	4.0%	Not available	0.5	-0.5	
Network size of relatives**					P<0.001
None	40.5%		-8.9	8.6	
1 or 2	35.3%		7.8	-7.5	
3 to 5	20.6%		0.6	-0.6	
More than 5	3.6%		0.5	-0.5	
Contact frequency with friends					P<0.001
No friends	6.7%	-4.9	0.3	3.9	
Less than once a month	26.7%	-6.5	4.8	1.3	
About once a month	21.7%	1.1	0.1	-1.2	
Several times a month	16.3%	7.7	0.8	-7.7	
About once a week	16.4%	3.8	-1.5	-1.8	
Several times a week	12.2%	-1.2	-4.5	5.5	
Contact frequency with relatives					P<0.001
No relatives	2.6%	-1.8	0.8	0.7	
Less than once a month	18.6%	-9.7	7.8	0.9	
About once a month	14.1%	-4.6	4.8	-0.5	
Several times a month	14.5%	2.1	4.3	-6.0	
About once a week	24.5%	2.8	-6.1	3.4	
Several times a week	25.8%	11.1	-11.6	1.5	
Participation in social activities					P<0.001
Never or not a member	81.8%	-13.8	4.9	7.4	
At least several times a year	8.8%	6.4	-2.0	-3.8	
Several times a month or more	9.4%	7.4	-2.9	-3.6	

*Country differences in mean cognitive function and proportion of social networks were compared using one-way analysis of variance and chi-squared tests, respectively.

**As data on social network size were not collected in the Czech Republic, a sub-sample was used for the analyses on social network size using data from Poland and Russia.
