# Supplementary material for: Social networks and cognitive function in older adults: findings from the HAPIEE study
Source: BMC Geriatr. 2021 Oct 18;21:570. doi: 10.1186/s12877-021-02531-0 (PMC8524850; doi:10.1186/s12877-021-02531-0)
Supplement: Supplementary file 3 — Additional file 3. Additional File 3. Cross-sectional associations of social network characteristics with specific cognitive functions. [file 12877_2021_2531_MOESM3_ESM.pdf]

Additional File 3. Cross-sectional associations of social network characteristics with specific cognitive functions

		Model 1*		Model 2†	
		b	95% CI	b	95% CI
Immediate word recall					
Network size - Friends	None	-0.01	-0.08, 0.05	0.00	-0.06, 0.06
	1 or 2	Reference		Reference	
	3 to 5	0.05	0.04, 0.13	0.06	-0.02, 0.13
	More than 5	0.18	0.05, 0.32	0.13	-0.00, 0.26
	P-trend	0.007		0.046	
Network size - Relatives	None	0.03	-0.03, 0.09	0.05	-0.01, 0.11
	1 or 2	Reference		Reference	
	3 to 5	0.02	-0.05, 0.10	0.04	-0.03, 0.11
	More than 5	0.04	-0.10, 0.19	0.01	-0.12, 0.15
	P-trend	0.807		0.528	
Contact frequency - Friends	No friends	-0.11	-0.18, -0.04	0.04	-0.05, 0.12
	Less than once a month	Reference		Reference	
	About once a month	0.05	-0.01, 0.11	0.03	-0.03, 0.08
	Several times a month	0.03	-0.04, 0.09	0.00	-0.06, 0.07
	About once a week	0.05	-0.01, 0.11	0.06	-0.00, 0.12
	Several times a week	-0.02	-0.10, 0.05	0.01	-0.05, 0.08
	P-trend	0.287		0.444	
Contact frequency - Relatives	No relatives	-0.01	-0.15, 0.12	-0.01	-0.14, 0.12
	Less than once a month	Reference		Reference	
	About once a month	-0.05	-0.12, 0.02	-0.04	-0.10, 0.03
	Several times a month	0.08	0.01, 0.16	0.07	0.00, 0.14
	About once a week	0.01	-0.05, 0.08	0.01	-0.05, 0.08
	Several times a week	0.03	-0.03, 0.10	0.01	-0.05, 0.07
	P-trend	0.147		0.452	
Participation in social activities	Never or not a member	Reference		Reference	
	At least several times a year	0.15	0.07, 0.22	0.03	-0.04, 0.10
	Several times a month or more	0.25	0.18, 0.32	0.13	0.06, 0.19
	P-trend	<0.001		<0.001	
Delayed word recall					
Network size - Friends	None	0.02	-0.05, 0.08	0.03	-0.03, 0.09
	1 or 2	Reference		Reference	
	3 to 5	0.05	-0.04, 0.13	0.05	-0.04, 0.13
	More than 5	-0.04	-0.18, 0.10	-0.09	-0.22, 0.05
	P-trend	0.954		0.429	
Network size - Relatives	None	0.01	-0.05, 0.07	0.02	-0.04, 0.08
	1 or 2	Reference		Reference	
	3 to 5	0.04	-0.04, 0.11	0.04	-0.03, 0.12
	More than 5	0.09	-0.06, 0.23	0.05	-0.09, 0.20
	P-trend	0.343		0.573	
Contact frequency - Friends	No friends	-0.06	-0.13, 0.01	0.04	-0.05, 0.13
	Less than once a month	Reference		Reference	
	About once a month	0.09	0.03, 0.15	0.07	0.01, 0.13
	Several times a month	-0.01	-0.07, 0.06	-0.02	-0.09, 0.05
	About once a week	0.01	-0.05, 0.08	0.02	-0.04, 0.09
	Several times a week	-0.04	-0.11, 0.04	-0.01	-0.08, 0.07
	P-trend	0.665		0.488	
Contact frequency – Relatives	No relatives	-0.04	-0.18, 0.10	-0.04	-0.18, 0.10
	Less than once a month	Reference		Reference	
	About once a month	-0.03	-0.11, 0.04	-0.02	-0.10, 0.05
	Several times a month	0.06	-0.02, 0.14	0.05	-0.03, 0.12
	About once a week	0.04	-0.03, 0.11	0.05	-0.02, 0.11
	Several times a week	0.05	-0.02, 0.12	0.03	-0.04, 0.10
	P-trend	0.028		0.098	

		Model 1*		Model 2†	
		**b**	**95% CI**	**b**	**95% CI**
Participation in social activities	Never or not a member	Reference		Reference	
	At least several times a year	0.08	0.00, 0.16	-0.01	-0.08, 0.07
	Several times a month or more	0.17	0.10, 0.25	0.08	0.01, 0.15
	P-trend	<0.001		0.063	
Verbal fluency					
Network size - Friends	None	0.01	-0.08, 0.11	0.03	-0.06, 0.12
	1 or 2	Reference		Reference	
	3 to 5	0.14	0.02, 0.27	0.13	0.01, 0.26
	More than 5	0.28	0.07, 0.49	0.21	0.00, 0.41
	P-trend	0.006		0.047	
Network size - Relatives	None	0.04	-0.06, 0.13	0.05	-0.04, 0.14
	1 or 2	Reference		Reference	
	3 to 5	0.05	-0.06, 0.16	0.05	-0.05, 0.16
	More than 5	0.34	0.12, 0.56	0.29	0.07, 0.50
	P-trend	0.157		0.304	
Contact frequency - Friends	No friends	-0.21	-0.32, -0.11	-0.04	-0.18, 0.10
	Less than once a month	Reference		Reference	
	About once a month	0.07	-0.03, 0.17	0.03	-0.06, 0.12
	Several times a month	0.09	-0.02, 0.20	0.06	-0.05, 0.16
	About once a week	0.03	-0.08 0.13	0.04	-0.07, 0.14
	Several times a week	-0.04	-0.16, 0.07	0.00	-0.11, 0.12
	P-trend	0.275		0.443	
Contact frequency – Relatives	No relatives	-0.03	-0.26, 0.19	-0.05	-0.27, 0.16
	Less than once a month	Reference		Reference	
	About once a month	-0.02	-0.14, 0.10	0.01	-0.11, 0.12
	Several times a month	0.15	0.03, 0.27	0.13	0.02, 0.25
	About once a week	0.06	-0.04, 0.17	0.08	-0.03, 0.18
	Several times a week	0.02	-0.09, 0.13	-0.01	-0.12, 0.09
	P-trend	0.333		0.705	
Participation in social activities	Never or not a member	Reference		Reference	
	At least several times a year	0.41	0.29, 0.53	0.25	0.14, 0.37
	Several times a month or more	0.35	0.23, 0.46	0.18	0.06, 0.29
	P-trend	<0.001		<0.001	
Processing speed					
Network size - Friends	None	0.02	-0.05, 0.09	0.02	-0.05, 0.09
	1 or 2	Reference		Reference	
	3 to 5	0.10	0.01, 0.19	0.10	0.01, 0.20
	More than 5	0.31	0.15, 0.47	0.27	0.12, 0.43
	P-trend	0.002		0.005	
Network size - Relatives	None	-0.07	-0.14, -0.00	-0.06	-0.13, 0.01
	1 or 2	Reference		Reference	
	3 to 5	0.07	-0.02, 0.15	0.08	-0.00, 0.16
	More than 5	0.15	-0.01, 0.32	0.14	-0.02, 0.30
	P-trend	<0.001		<0.001	
Contact frequency - Friends	No friends	-0.14	-0.21, -0.06	0.00	-0.10, 0.10
	Less than once a month	Reference		Reference	
	About once a month	0.04	-0.03, 0.10	0.02	-0.05, 0.09
	Several times a month	-0.01	-0.09, 0.06	-0.02	-0.09, 0.06
	About once a week	-0.03	-0.11, 0.04	-0.01	-0.09, 0.06
	Several times a week	-0.02	-0.10, 0.06	0.03	-0.05, 0.11
	P-trend	0.963		0.821	
Contact frequency – Relatives	No relatives	0.03	-0.12, 0.19	0.04	-0.11, 0.20
	Less than once a month	Reference		Reference	
	About once a month	0.05	-0.04, 0.13	0.05	-0.03, 0.13
	Several times a month	0.12	0.04, 0.20	0.11	0.03, 0.19
	About once a week	0.08	0.01, 0.15	0.08	0.01, 0.16
	Several times a week	0.11	0.03, 0.18	0.09	0.02, 0.16
	P-trend	0.006		0.019	

		Model 1*		Model 2†	
		**b**	**95% CI**	**b**	**95% CI**
Participation in social activities	Never or not a member	Reference		Reference	
	At least several times a year	0.11	0.02, 0.19	0.01	-0.07, 0.09
	Several times a month or more	0.13	0.04, 0.21	0.02	-0.06, 0.10
	P-trend	<0.001		0.574	

*Adjusted for country, age and sex.

†Adjusted for country, age, sex, education, household amenities, work status, marital status, smoking status, alcohol drinking frequency, alcohol intake, physical activity, self-rated health, number of chronic diseases and depressive symptoms.
