# Supplementary material for: Social networks and cognitive function in older adults: findings from the HAPIEE study
Source: BMC Geriatr. 2021 Oct 18;21:570. doi: 10.1186/s12877-021-02531-0 (PMC8524850; doi:10.1186/s12877-021-02531-0)
Supplement: Supplementary file 4 — Additional file 4. Prospective associations of social network characteristics with specific cognitive functions. [file 12877_2021_2531_MOESM4_ESM.pdf]

Additional File 4. Prospective associations of social network characteristics with specific cognitive functions

		Model 1*		Model 2†		Model 3‡	
		b	95% CI	b	95% CI	b	95% CI
Immediate word recall							
Network size - Friends	None	-0.03	-0.09, 0.04	-0.03	-0.09, 0.03	-0.02	-0.08, 0.04
	1 or 2	Reference		Reference		Reference	
	3 to 5	0.02	-0.07, 0.11	-0.01	-0.09, 0.07	0.01	-0.07, 0.09
	More than 5	0.12	-0.03, 0.26	0.03	-0.11, 0.17	0.02	-0.12, 0.15
	P-trend	0.065		0.378		0.444	
Network size - Relatives	None	-0.04	-0.10, 0.03	-0.05	-0.11, 0.01	-0.04	-0.10, 0.02
	1 or 2	Reference		Reference		Reference	
	3 to 5	-0.02	-0.10, 0.05	-0.03	-0.10, 0.04	-0.02	-0.09, 0.05
	More than 5	0.08	-0.07, 0.24	0.07	-0.07, 0.21	0.05	-0.08, 0.19
	P-trend	0.249		0.133		0.237	
Contact frequency - Friends	No friends	-0.18	-0.28, -0.09	-0.14	-0.23, -0.05	-0.05	-0.13, 0.04
	Less than once a month	Reference		Reference		Reference	
	About once a month	0.05	-0.01, 0.12	0.02	-0.03, 0.08	0.02	-0.04, 0.07
	Several times a month	-0.01	-0.08, 0.06	-0.02	-0.08, 0.04	-0.02	-0.09, 0.04
	About once a week	0.01	-0.06, 0.09	-0.01	-0.08, 0.05	0.01	-0.05, 0.07
	Several times a week	-0.03	-0.11, 0.05	-0.02	-0.09, 0.05	0.02	-0.05, 0.09
	P-trend	0.335		0.585		0.389	
Contact frequency - Relatives	No relatives	-0.06	-0.21, 0.09	-0.07	-0.20, 0.07	-0.05	-0.18, 0.08
	Less than once a month	Reference		Reference		Reference	
	About once a month	-0.09	-0.16, -0.01	-0.06	-0.13, 0.01	-0.06	-0.12, 0.01
	Several times a month	0.07	-0.01, 0.15	0.03	-0.04, 0.10	0.03	-0.04, 0.10
	About once a week	-0.02	-0.10, 0.05	-0.03	-0.09, 0.04	-0.02	-0.09, 0.04
	Several times a week	-0.01	-0.08, 0.07	-0.02	-0.08, 0.04	-0.03	-0.10, 0.03
	P-trend	0.605		0.953		0.570	
Participation in social activities	Never or not a member	Reference		Reference		Reference	
	At least several times a year	0.17	0.09, 0.25	0.10	0.03, 0.18	0.03	-0.04, 0.10
	Several times a month or more	0.20	0.13, 0.28	0.09	0.22, 0.16	0.01	-0.06, 0.08
	P-trend	<0.001		0.002		0.665	
Delayed word recall							
Network size - Friends	None	-0.03	-0.10, 0.03	-0.04	-0.10, 0.02	-0.03	-0.10, 0.03
	1 or 2	Reference		Reference		Reference	
	3 to 5	0.03	-0.06, 0.12	0.01	-0.07, 0.10	0.02	-0.06, 0.10
	More than 5	0.07	-0.08, 0.21	0.09	-0.05, 0.22	0.05	-0.08, 0.19
	P-trend	0.057		0.038		0.086	
Network size - Relatives	None	-0.01	-0.08, 0.05	-0.02	-0.08, 0.04	-0.01	-0.07, 0.05
	1 or 2	Reference		Reference		Reference	
	3 to 5	0.01	-0.07, 0.09	-0.00	-0.07, 0.07	0.00	-0.07, 0.07
	More than 5	0.04	-0.11, 0.19	0.02	-0.12, 0.16	0.01	-0.13, 0.15
	P-trend	0.452		0.396		0.644	
Contact frequency - Friends	No friends	-0.16	-0.26, -0.06	-0.13	-0.22, -0.04	-0.06	-0.15, 0.03
	Less than once a month	Reference		Reference		Reference	
	About once a month	0.05	-0.02, 0.12	0.01	-0.05, 0.07	0.00	-0.06, 0.06
	Several times a month	-0.01	-0.08, 0.06	-0.01	-0.07, 0.06	-0.02	-0.08, 0.05
	About once a week	0.05	-0.03, 0.12	0.04	-0.03, 0.10	0.05	-0.02, 0.11
	Several times a week	-0.03	-0.11, 0.05	-0.02	-0.09, 0.05	0.01	-0.06, 0.08
	P-trend	0.206		0.127		0.139	
Contact frequency – Relatives	No relatives	-0.03	-0.18, 0.12	-0.02	-0.16, 0.11	-0.02	-0.15, 0.12
	Less than once a month	Reference		Reference		Reference	
	About once a month	-0.07	-0.15, 0.01	-0.05	-0.13, 0.02	-0.05	-0.12, 0.02
	Several times a month	0.06	-0.02, 0.14	0.04	-0.03, 0.11	0.03	-0.04, 0.11
	About once a week	-0.01	-0.08, 0.06	-0.02	-0.09, 0.04	-0.02	-0.08, 0.04
	Several times a week	-0.00	-0.08, 0.07	-0.02	-0.09, 0.04	-0.03	-0.10, 0.03
	P-trend	0.655		0.765		0.464	

		Model 1[*]		Model 2[†]		Model 3[‡]	
		b	95% CI	b	95% CI	b	95% CI
Participation in social activities	Never or not a member	Reference		Reference		Reference	
	At least several times a year	0.14	0.06, 0.22	0.11	0.03, 0.18	0.04	-0.03, 0.12
	Several times a month or more	0.18	0.10, 0.26	0.10	0.03, 0.18	0.04	-0.04, 0.11
	P-trend	<0.001		<0.001		0.202	
Verbal fluency							
Network size - Friends	None	-0.00	-0.07, 0.07	0.00	-0.06, 0.06	-0.01	-0.07, 0.05
	1 or 2	Reference		Reference		Reference	
	3 to 5	0.01	-0.08, 0.10	-0.03	-0.11, 0.06	-0.02	-0.11, 0.06
	More than 5	0.12	-0.03, 0.28	0.05	-0.10, 0.19	0.02	-0.12, 0.16
	P-trend	0.261		0.923		0.875	
Network size - Relatives	None	0.07	0.00, 0.14	0.06	-0.00, 0.12	0.06	-0.00, 0.12
	1 or 2	Reference		Reference		Reference	
	3 to 5	0.05	-0.03, 0.13	0.04	-0.04, 0.11	0.04	-0.03, 0.12
	More than 5	0.10	-0.06, 0.26	0.01	-0.13, -0.16	-0.00	-0.15, 0.14
	P-trend	0.677		0.324		0.350	
Contact frequency - Friends	No friends	-0.04	-0.14, 0.06	0.03	-0.06, 0.12	0.09	-0.00, 0.18
	Less than once a month	Reference		Reference		Reference	
	About once a month	0.07	-0.00, 0.13	0.03	-0.03, 0.09	0.04	-0.02, 0.10
	Several times a month	0.02	-0.06, 0.09	-0.03	-0.09, 0.04	-0.01	-0.07, 0.06
	About once a week	0.02	-0.05, 0.10	0.00	-0.07, 0.07	0.04	-0.03, 0.10
	Several times a week	-0.04	-0.12, 0.04	-0.03	-0.10, 0.05	0.01	-0.06, 0.09
	P-trend	0.621		0.241		0.629	
Contact frequency – Relatives	No relatives	0.09	-0.07, 0.24	0.09	-0.05, 0.23	0.09	-0.05, 0.22
	Less than once a month	Reference		Reference		Reference	
	About once a month	-0.05	-0.14, 0.03	-0.04	-0.12, 0.03	-0.04	-0.11, 0.04
	Several times a month	-0.03	-0.11, 0.05	-0.07	-0.15, 0.00	-0.07	-0.14, 0.00
	About once a week	-0.12	-0.20, -0.05	-0.14	-0.20, -0.07	-0.13	-0.19, -0.06
	Several times a week	0.03	-0.05, 0.10	0.02	-0.05, 0.09	0.00	-0.06, 0.07
	P-trend	0.637		0.271		0.156	
Participation in social activities	Never or not a member	Reference		Reference		Reference	
	At least several times a year	0.17	0.01, 0.26	0.06	-0.02, 0.13	0.00	-0.07, 0.08
	Several times a month or more	0.18	0.10, 0.26	0.08	0.01, 0.16	0.01	-0.06, 0.09
	P-trend	<0.001		0.012		0.708	
Processing speed							
Network size - Friends	None	-0.03	-0.10, 0.03	-0.03	-0.09, 0.03	-0.04	-0.10, 0.02
	1 or 2	Reference		Reference		Reference	
	3 to 5	-0.00	-0.09, 0.08	-0.04	-0.12, 0.04	-0.03	-0.11, 0.05
	More than 5	-0.03	-0.18, 0.11	-0.14	-0.27, -0.00	-0.15	-0.28, -0.02
	P-trend	0.558		0.428		0.535	
Network size - Relatives	None	-0.00	-0.06, 0.06	0.02	-0.04, 0.08	0.03	-0.03, 0.08
	1 or 2	Reference		Reference		Reference	
	3 to 5	-0.00	-0.08, 0.07	-0.02	-0.09, 0.05	-0.01	-0.08, 0.06
	More than 5	0.09	-0.06, 0.24	0.03	-0.11, 0.17	0.03	-0.11, 0.17
	P-trend	0.560		0.444		0.373	
Contact frequency - Friends	No friends	-0.15	-0.24, -0.05	-0.10	-0.19, -0.01	-0.02	-0.10, 0.07
	Less than once a month	Reference		Reference		Reference	
	About once a month	0.07	0.01, 0.14	0.05	-0.01, 0.11	0.05	-0.01, 0.11
	Several times a month	-0.01	-0.08, 0.06	-0.00	-0.07, 0.06	-0.01	-0.07, 0.06
	About once a week	0.01	-0.07, 0.08	0.01	-0.05, 0.08	0.03	-0.04, 0.09
	Several times a week	-0.06	-0.14, 0.02	-0.02	-0.12, 0.02	-0.01	-0.08, 0.06
	P-trend	0.933		0.919		0.986	
Contact frequency – Relatives	No relatives	-0.04	-0.19, 0.11	-0.07	-0.21, 0.07	-0.05	-0.19, 0.08
	Less than once a month	Reference		Reference		Reference	
	About once a month	-0.08	-0.16, -0.00	-0.09	-0.17, -0.02	-0.09	-0.16, -0.02
	Several times a month	0.03	-0.05, 0.11	-0.01	-0.08, 0.06	-0.02	-0.09, 0.05
	About once a week	-0.01	-0.08, 0.06	-0.03	-0.10, 0.03	-0.03	-0.10, 0.03
	Several times a week	0.01	-0.06, 0.08	-0.03	-0.10, 0.04	-0.04	-0.11, 0.02
	P-trend	0.252		0.945		0.724	

		Model 1*		Model 2†		Model 3‡	
		b	95% CI	b	95% CI	b	95% CI
Participation in social activities	Never or not a member	Reference		Reference		Reference	
	At least several times a year	0.12	0.04, 0.20	0.08	0.01, 0.16	0.01	-0.06, 0.08
	Several times a month or more	0.20	0.12, 0.28	0.15	0.08, 0.23	0.07	-0.00, 0.14
	P-trend	<0.001		<0.001		0.084	

*Adjusted for country, age and sex.

†Adjusted for country, age, sex and baseline cognitive function.

‡Adjusted for country, age, sex, baseline cognitive function, education, household amenities, work status, marital status, smoking status, alcohol drinking frequency, alcohol intake, physical activity, self-rated health, number of chronic diseases and depressive symptoms.
