# Supplementary material for: Social networks and cognitive function in older adults: findings from the HAPIEE study
Source: BMC Geriatr. 2021 Oct 18;21:570. doi: 10.1186/s12877-021-02531-0 (PMC8524850; doi:10.1186/s12877-021-02531-0)
Supplement: Supplementary file 5 — Additional file 5. Comparison of baseline study characteristics between complete (n=6,691 and n=4,624) and incomplete (n=11,920) cases. [file 12877_2021_2531_MOESM5_ESM.pdf]

Additional File 5. Comparison of baseline study characteristics between complete (n=6,691 and n=4,624) and incomplete (n=11,920) cases

% or Mean (SD) for measures at baseline (2002/2005)	Complete cases*	Incomplete cases	P-value of sample differences**
Immediate recall (0-30)	20.8 (4.3)	20.3 (4.4)	P<0.001
Delayed recall (0-10)	7.0 (1.9)	6.8 (2.0)	P<0.001
Verbal fluency	20.6 (6.9)	20.0 (6.8)	P<0.001
Processing speed (0-65)	17.7 (5.5)	17.3 (5.5)	P<0.001
Country			P<0.001
Czech Republic	30.9%	30.2%	
Poland	34.0%	33.8%	
Russia	35.1%	36.0%	
Age (years)	62.2 (6.0)	62.4 (5.9)	P<0.001
Female	53.7%	53.0%	P<0.001
Network size of friends			P<0.001
None	46.5%	50.6%	
1 or 2	35.3%	32.8%	
3 to 5	14.3%	13.0%	
More than 5	4.0%	3.5%	
Network size of relatives			
None	40.5%	43.6%	
1 or 2	35.3%	34.7%	
3 to 5	20.6%	18.5%	
More than 5	3.6%	3.3%	
Contact frequency with friends			P<0.001
No friends	6.7%	8.0%	
Less than once a month	26.7%	29.1%	
About once a month	21.7%	20.4%	
Several times a month	16.3%	15.2%	
About once a week	16.4%	15.6%	
Several times a week	12.2%	11.8%	
Contact frequency with relatives			
No relatives	2.6%	2.9%	
Less than once a month	18.6%	21.1%	
About once a month	14.1%	14.3%	
Several times a month	14.5%	13.8%	
About once a week	24.5%	23.4%	
Several times a week	25.8%	24.6%	
Participation in social activities			P<0.001
Never or not a member	81.8%	84.3%	
At least several times a year	8.8%	7.7%	
Several times a month or more	9.4%	8.0%	
Educational level			P<0.001
Primary or lower	9.6%	13.4%	
Vocational	26.2%	26.7%	
Secondary	37.0%	35.8%	
University	27.3%	24.1%	
Number of household amenities (0-12)	6.3 (2.2)	6.1(2.2)	P<0.001
Not working	64.6%	67.1%	P<0.001
Not partnered	25.6%	27.1%	P<0.001
Smoking status			P<0.001
Current	20.4%	22.7%	
Former	25.2%	25.2%	
Never	54.3%	52.1%	
Alcohol drinking frequency			P<0.001
Never	21.5%	24.0%	
<1/month	29.9%	30.0%	
1-3/month	20.3%	19.4%	
1-4/week	21.9%	20.6%	
More than 5/week	6.4%	6.0%	
Alcohol intake per week (grams)	2580.7 (6134.5)	2544.4 (6386.9)	P<0.001

**% or Mean (SD) for measures at baseline (2002/2005)**	**Complete cases***	**Incomplete cases**	**P-value of sample differences****
Physical activity per week (hours)	21.2 (14.3)	20.1 (14.2)	P<0.001
Self-rated health			P<0.001
Very poor or poor	14.5%	16.9%	
Fair	59.6%	59.4%	
Very good or good	26.0%	23.7%	
Number of chronic diseases (0-4)	3.7 (0.6)	3.6 (0.7)	P<0.001
CES-D 20 score (0-60)	10.9 (7.9)	11.1 (8.1)	P<0.001

Note: Covariate baseline data on age, sex, and country were complete for all participants.

*Complete case estimates are based on the analytic sample for frequency of contact with social network members and participation in social activities (n=6,691), with exception to baseline frequencies on social network size which was calculated using the sub-sample of participants with these data (n=4,624).

**Proportion and mean differences between complete and incomplete cases were compared using chi-squared tests and t-tests.
