# Supplementary material for: Social networks and cognitive function in older adults: findings from the HAPIEE study
Source: BMC Geriatr. 2021 Oct 18;21:570. doi: 10.1186/s12877-021-02531-0 (PMC8524850; doi:10.1186/s12877-021-02531-0)
Supplement: Supplementary file 6 — Additional file 6. Comparison of country, age, and sex adjusted cross-sectional associations between social network characteristics and global cognition among complete (n=6,691 and n=4,624) and incomplete cases due to loss to follow-up (n=10,440 and 7,365). [file 12877_2021_2531_MOESM6_ESM.pdf]

Additional File 6. Comparison of country, age, and sex adjusted cross-sectional associations between social network characteristics and global cognition among complete (n=6,691 and n=4,624) and incomplete cases due to loss to follow-up (n=10,440 and 7,365)

**Social network measure**		**Complete cases**		**Incomplete cases***	
		**b**	**95% CI**	**b**	**95% CI**
Network size – Friends	None	0.01	-0.05, 0.06	-0.03	-0.07, 0.01
	1 or 2	Reference		Reference	
	3 to 5	0.08	0.01, 0.15	0.09	0.04, 0.15
	More than 5	0.18	0.06, 0.30	0.17	0.07, 0.27
	P-trend	0.003		<0.001	
Network size – Relatives	None	0.00	-0.05, 0.05	0.00	-0.04, 0.04
	1 or 2	Reference		Reference	
	3 to 5	0.04	-0.02, 0.11	0.05	0.00, 0.10
	More than 5	0.16	0.03, 0.28	0.13	0.03, 0.23
	P-trend	0.010		0.010	
Contact frequency - Friends	No friends	-0.12	-0.30, -0.05	-0.13	-0.19, -0.07
	Less than once a month	Reference		Reference	
	About once a month	0.06	0.01, 0.12	0.07	0.03, 0.11
	Several times a month	0.02	-0.03, 0.08	0.08	0.03, 0.13
	About once a week	0.01	-0.04, 0.07	0.04	-0.01, 0.09
	Several times a week	-0.03	-0.10, 0.03	0.01	-0.04, 0.07
	P-trend	0.490		0.001	
Contact frequency - Relatives	No relatives	-0.01	-0.13, 0.11	-0.08	-0.18, 0.01
	Less than once a month	Reference		Reference	
	About once a month	-0.02	-0.08, 0.05	0.02	-0.03, 0.07
	Several times a month	0.10	0.04, 0.17	0.10	0.05, 0.15
	About once a week	0.05	-0.01, 0.11	0.07	0.03, 0.12
	Several times a week	0.05	-0.01, 0.11	0.04	-0.01, 0.09
	P-trend	0.017		0.002	
Participation in social activities	Never or not a member	Reference		Reference	
	At least several times a year	0.19	0.19	0.21	0.16, 0.27
	Several times a month or more	0.22	0.22	0.28	0.23, 0.34
	P-trend	<0.001		<0.001	

*Incomplete case analyses due to loss to follow-up were performed on participants with baseline data on cognitive function (n=11,920) as well as social network characteristics and covariates. This amounted to 10,440 participants for the analyses on frequency of contact with social network members / participation in social activities and 7,365 participants for the analyses on social network size.
