# Supplementary material for: Social networks and cognitive function in older adults: findings from the HAPIEE study
Source: BMC Geriatr. 2021 Oct 18;21:570. doi: 10.1186/s12877-021-02531-0 (PMC8524850; doi:10.1186/s12877-021-02531-0)
Supplement: Supplementary file 7 — Additional file 7. Cross-sectional associations of social network characteristics with global cognitive function in participants re-examined between 3-4 years of follow-up. [file 12877_2021_2531_MOESM7_ESM.pdf]

Additional File 7. Cross-sectional associations of social network characteristics with global cognitive function in participants re-examined between 3-4 years of follow-up

Social network measure	Model 1*		Model 2†	
	b	95% CI	b	95% CI
**Network size of friends‡**				
None	-0.02	-0.08, 0.03	-0.00	-0.06, 0.05
1 or 2	Reference		Reference	
3 to 5	0.07	-0.01, 0.14	0.07	-0.01, 0.14
More than 5	0.19	0.06, 0.31	0.14	0.02, 0.26
P-trend	<0.001		0.011	
**Network size of relatives‡**				
None	-0.03	-0.08, 0.03	-0.01	-0.07, 0.04
1 or 2	Reference		Reference	
3 to 5	0.05	-0.02, 0.11	0.05	-0.01, 0.11
More than 5	0.14	0.01, 0.27	0.11	-0.02, 0.23
P-trend	0.004		0.017	
**Contact frequency with friends§**				
No friends	-0.13	-0.22, -0.04	0.01	-0.07, 0.09
Less than once a month	Reference		Reference	
About once a month	0.07	0.01, 0.12	0.04	-0.01, 0.09
Several times a month	0.03	-0.03, 0.10	0.02	-0.04, 0.07
About once a week	0.03	-0.03, 0.10	0.05	-0.01, 0.11
Several times a week	-0.02	-0.09, 0.04	0.02	-0.05, 0.08
P-trend	0.228		0.307	
**Contact frequency with relatives§**				
No relatives	-0.01	-0.14, 0.12	-0.02	-0.14, 0.10
Less than once a month	Reference		Reference	
About once a month	0.00	-0.07, 0.07	0.00	-0.06, 0.07
Several times a month	0.12	0.05, 0.19	0.10	-0.04, 0.16
About once a week	0.07	0.01, 0.13	0.07	0.01, 0.12
Several times a week	0.06	0.00, 0.13	0.04	-0.02, 0.09
P-trend	0.007		0.049	
**Participation in social activities§**				
Never or not a member	Reference		Reference	
At least several times a year	0.18	0.11, 0.25	0.07	0.00, 0.13
Several times a month or more	0.21	0.14, 0.27	0.09	0.02, 0.15
P-trend	<0.001		0.002	

*Adjusted for country, age and sex.

†Adjusted for country, age, sex, education, household amenities, work status, marital status, smoking status, alcohol drinking frequency, alcohol intake, physical activity, self-rated health, number of chronic diseases and depressive symptoms.

‡Results are based on 84.3% (n=3,900) of participants from the analytic sample for social network size who were re-examined between 3-4 years of follow-up.

§Results are based on 87.7% (n=5,868) of participants from the analytic sample for contact frequency and social activities who were re-examined between 3-4 years of follow-up.
