# Supplementary material for: Social networks and cognitive function in older adults: findings from the HAPIEE study
Source: BMC Geriatr. 2021 Oct 18;21:570. doi: 10.1186/s12877-021-02531-0 (PMC8524850; doi:10.1186/s12877-021-02531-0)
Supplement: Supplementary file 8 — Additional file 8. Prospective associations of social network characteristics with global cognitive function in participants re-examined between 3-4 years of follow-up. [file 12877_2021_2531_MOESM8_ESM.pdf]

Additional File 8. Prospective associations of social network characteristics with global cognitive function in participants re-examined between 3-4 years of follow-up

**Social network measure**	**Model 1***		**Model 2†**		**Model 3‡**	
	**b**	**95% CI**	**b**	**95% CI**	**b**	**95% CI**
**Network size of friends§**						
None	-0.03	-0.08, 0.03	-0.02	-0.06, 0.03	-0.01	-0.06, 0.03
1 or 2	Reference		Reference		Reference	
3 to 5	-0.00	-0.07, 0.07	-0.03	-0.10, 0.03	-0.02	-0.07, 0.04
More than 5	0.06	-0.06, 0.18	-0.02	-0.12, 0.09	-0.02	-0.12, 0.08
P-trend	0.156		0.744		0.945	
**Network size of relatives§**						
None	0.00	-0.05, 0.05	0.01	-0.03, 0.06	0.02	-0.03, 0.06
1 or 2	Reference		Reference		Reference	
3 to 5	0.03	-0.04, 0.09	0.01	-0.05, 0.06	0.01	-0.04, 0.06
More than 5	0.08	-0.05, 0.20	0.01	-0.09, 0.12	0.00	-0.10, 0.11
P-trend	0.233		0.834		0.706	
**Contact frequency with friends¶**						
No friends	-0.15	-0.23, -0.07	-0.08	-0.15, -0.01	-0.02	-0.08, 0.05
Less than once a month	Reference		Reference		Reference	
About once a month	0.06	0.01, 0.11	0.02	-0.02, 0.07	0.02	-0.02, 0.06
Several times a month	0.00	-0.05, 0.06	-0.01	-0.06, 0.03	-0.01	-0.06, 0.03
About once a week	0.03	-0.03, 0.08	0.01	-0.04, 0.06	0.03	-0.02, 0.07
Several times a week	-0.04	-0.10, 0.02	-0.03	-0.08, 0.02	0.01	-0.04, 0.06
P-trend	0.130		0.902		0.477	
**Contact frequency with relatives¶**						
No relatives	-0.04	-0.16, 0.08	-0.04	-0.14, 0.06	-0.05	-0.15, 0.05
Less than once a month	Reference		Reference		Reference	
About once a month	-0.06	-0.12, 0.00	-0.06	-0.11, -0.01	-0.06	-0.11, -0.01
Several times a month	0.03	-0.03, 0.10	-0.03	-0.08, 0.03	-0.03	-0.08, 0.03
About once a week	-0.02	-0.08, 0.03	-0.06	-0.11, -0.01	-0.05	-0.10, -0.01
Several times a week	0.01	-0.04, 0.07	-0.02	-0.07, 0.03	-0.03	-0.08, 0.01
P-trend	0.667		0.596		0.278	
**Participation in social activities¶**						
Never or not a member	Reference		Reference		Reference	
At least several times a year	0.25	0.16, 0.35	0.17	0.08, 0.25	0.02	-0.03, 0.07
Several times a month or more	0.16	0.07, 0.25	0.06	-0.02, 0.15	0.01	-0.04, 0.06
P-trend	<0.001		0.003		0.568	

*Adjusted for country, age and sex.
†Adjusted for country, age, sex and baseline global cognitive function.
‡Adjusted for country, age, sex, baseline global cognitive function, education, household amenities, work status, marital status, smoking status, alcohol drinking frequency, alcohol intake, physical activity, self-rated health, number of chronic diseases and depressive symptoms.
§ Results are based on 84.3% (n=3,900) of participants from the analytic sample for social network size who were re-examined between 3-4 years of follow-up.
¶Results are based on 87.7% (n=5,868) of participants from the analytic sample for contact frequency and social activities who were re-examined between 3-4 years of follow-up.
