# Supplementary material for: Social networks and cognitive function in older adults: findings from the HAPIEE study
Source: BMC Geriatr. 2021 Oct 18;21:570. doi: 10.1186/s12877-021-02531-0 (PMC8524850; doi:10.1186/s12877-021-02531-0)
Supplement: Supplementary file 9 — Additional file 9. Cross-sectional associations between social network characteristics and global cognitive function with Bonferroni corrected p-values. [file 12877_2021_2531_MOESM9_ESM.pdf]

Additional File 9. Cross-sectional associations between social network characteristics and global cognitive function with Bonferroni corrected p-values

Social network measure	Model 1*			Model 2†		
	b	95% CI	P-value‡	b	95% CI	P-value‡
**Network size of friends**						
None	0.01	-0.05, 0.06	1.000	0.02	-0.03, 0.07	1.000
1 or 2	Reference			Reference		
3 to 5	0.08	0.01, 0.15	1.000	0.08	0.01, 0.14	0.401
More than 5	**0.18**	**0.06, 0.30**	**0.015**	0.13	0.02, 0.24	0.412
P-trend	0.003			0.036		
**Network size of relatives**						
None	0.00	-0.05, 0.05	1.000	0.01	-0.04, 0.06	1.000
1 or 2	Reference			Reference		
3 to 5	0.04	-0.02, 0.11	0.840	0.05	0.00, 0.11	1.000
More than 5	0.16	0.03, 0.28	0.070	0.12	0.01, 0.24	0.614
P-trend	0.010			0.074		
**Contact frequency with friends**						
No friends	**-0.17**	**-0.20, -0.05**	**0.010**	0.01	-0.06, 0.09	1.000
Less than once a month	Reference			Reference		
About once a month	0.06	0.01, 0.12	0.105	0.04	-0.01, 0.08	1.000
Several times a month	0.02	-0.03, 0.08	1.000	0.01	-0.05, 0.06	1.000
About once a week	0.01	-0.04, 0.07	1.000	0.03	-0.03, 0.08	1.000
Several times a week	-0.03	-0.10, 0.03	1.000	0.01	-0.05, 0.07	1.000
P-trend	0.490			0.655		
**Contact frequency with relatives**						
No relatives	-0.01	-0.13, 0.11	1.000	-0.01	-0.13, 0.10	1.000
Less than once a month	Reference			Reference		
About once a month	-0.02	-0.08, 0.05	1.000	0.00	-0.06, 0.06	1.000
Several times a month	**0.10**	**0.04, 0.17**	**0.010**	**0.09**	**0.03, 0.15**	**0.050**
About once a week	0.05	-0.01, 0.11	0.475	0.06	0.00, 0.11	0.696
Several times a week	0.05	-0.01, 0.11	0.405	0.03	-0.03, 0.08	1.000
P-trend	0.017			0.091		
**Participation in social activities**						
Never or not a member	Reference			Reference		
At least several times a year	**0.19**	**0.12, 0.25**	**<0.001**	0.07	0.01, 0.13	0.320
Several times a month or more	**0.22**	**0.16, 0.29**	**<0.001**	**0.10**	**0.04, 0.16**	**0.016**
P-trend	<0.001			<0.001		

*Adjusted for country, age and sex.

†Adjusted for country, age, sex, education, household amenities, work status, marital status, smoking status, alcohol drinking frequency, alcohol intake, physical activity, self-rated health, number of chronic diseases and depressive symptoms.

‡P-values after Bonferroni correction for multiple testing. Emboldened estimates were statistically significant (p-value<0.05).
