# Supplementary material for: Social networks and cognitive function in older adults: findings from the HAPIEE study
Source: BMC Geriatr. 2021 Oct 18;21:570. doi: 10.1186/s12877-021-02531-0 (PMC8524850; doi:10.1186/s12877-021-02531-0)
Supplement: Supplementary file 10 — Additional file 10. Prospective associations between social network characteristics and global cognitive function with Bonferroni corrected p-values. [file 12877_2021_2531_MOESM10_ESM.pdf]

Additional File 10. Prospective associations between social network characteristics and global cognitive function with Bonferroni corrected p-values

Social network measure	Model 1*			Model 2†			Model 3‡		
	b	95% CI	P-value§	b	95% CI	P-value§	b	95% CI	P-value§
**Network size of friends**									
None	-0.02	-0.07, 0.03	1.000	-0.02	-0.07, 0.02	1.000	-0.03	-0.07, 0.02	1.000
1 or 2	Reference			Reference			Reference		
3 to 5	0.01	-0.05, 0.08	1.000	-0.03	-0.08, 0.03	1.000	-0.01	-0.07, 0.04	1.000
More than 5	0.06	-0.05, 0.17	1.000	-0.02	-0.11, 0.08	1.000	-0.03	-0.12, 0.06	1.000
P-trend	0.095			0.787			0.643		
**Network size of relatives**									
None	0.00	-0.05, 0.05	1.000	0.00	-0.04, 0.04	1.000	0.00	-0.04, 0.04	1.000
1 or 2	Reference			Reference			Reference		
3 to 5	0.01	-0.05, 0.07	1.000	-0.01	-0.06, 0.04	1.000	0.00	-0.05, 0.04	1.000
More than 5	0.08	-0.03, 0.19	0.860	0.01	-0.09, 0.11	1.000	0.00	-0.09, 0.10	1.000
P-trend	0.425			0.815			0.828		
**Contact frequency with friends**									
No friends	**-0.13**	**-0.20, -0.06**	**<0.001**	-0.07	-0.13, -0.01	0.150	-0.01	-0.07, 0.05	1.000
Less than once a month	Reference			Reference			Reference		
About once a month	0.06	0.01, 0.11	0.075	0.02	-0.02, 0.06	0.942	0.02	-0.02, 0.06	1.000
Several times a month	0.00	-0.05, 0.05	1.000	-0.02	-0.07, 0.02	1.000	-0.02	-0.06, 0.03	1.000
About once a week	0.02	-0.03, 0.07	1.000	0.00	-0.04, 0.05	1.000	0.03	-0.02, 0.07	1.000
Several times a week	-0.04	-0.10, 0.02	0.925	-0.03	-0.08, 0.02	1.000	0.01	-0.04, 0.06	1.000
P-trend	0.580			0.827			0.535		
**Contact frequency with relatives**									
No relatives	-0.01	-0.12, 0.10	1.000	-0.01	-0.11, 0.08	1.000	-0.01	-0.10, 0.08	1.000
Less than once a month	Reference			Reference			Reference		
About once a month	-0.07	-0.13, -0.01	0.070	-0.06	-0.11, -0.01	0.054	-0.05	-0.10, -0.01	1.000
Several times a month	0.03	-0.03, 0.09	1.000	-0.02	-0.07, 0.13	1.000	-0.01	-0.06, 0.03	1.000
About once a week	-0.04	-0.09, 0.01	0.620	**-0.06**	**-0.11, -0.02**	**0.020**	-0.05	-0.09, -0.01	1.000
Several times a week	0.00	-0.04, 0.06	1.000	-0.02	-0.06, 0.03	1.000	-0.03	-0.07, 0.01	1.000
P-trend	0.583			0.405			0.203		
**Participation in social activities**									
Never or not a member	Reference			Reference			Reference		
At least several times a year	**0.15**	**0.09, 0.21**	**<0.001**	0.06	0.01, 0.11	0.075	0.01	-0.04, 0.06	1.000
Several times a month or more	**0.19**	**0.13, 0.25**	**<0.001**	**0.08**	**0.03, 0.13**	**0.004**	0.03	-0.02, 0.08	1.000
P-trend	<0.001			<0.001			0.233		

*Adjusted for country, age and sex.

†Adjusted for country, age, sex and baseline global cognitive function.

‡Adjusted for country, age, sex, baseline global cognitive function, education, household amenities, work status, marital status, smoking status, alcohol drinking frequency, alcohol intake, physical activity, self-rated health, number of chronic diseases and depressive symptoms.

§P-values after Bonferroni correction for multiple testing. Emboldened estimates were statistically significant (p-value<0.05).
